# Zen meditation neutralizes emotional evaluation, but not implicit affective processing of words

**DOI:** 10.1371/journal.pone.0229310

**Published:** 2020-02-19

**Authors:** Larissa Lusnig, Ralph Radach, Christina J. Mueller, Markus J. Hofmann

**Affiliations:** 1 Department of Psychology, University of Wuppertal, Wuppertal, Germany; 2 Methods and Evaluation, International Psychoanalytic University, Berlin, Germany; University of California, San Francisco, UNITED STATES

## Abstract

There is ample evidence that meditation can regulate emotions. It is questionable, however, whether meditation can down-regulate sensitivity to emotional experience in high-level cognitive representations such as words. The present study shows that adept Zen meditators rated the emotional valence of (low-arousal) positive and (high- and low-arousal) negative nouns significantly more neutral after a meditation session, while there was no change of valence ratings after a comparison intervention in the comparison group. Because the Zen group provided greater “openness to experience” and lower „need for achievement and performance” in the “Big Five” personality assessment, we used these scores as covariates for all analyses. We found no differential emotion effects of Zen meditation during lexical decision, but we replicated the slow-down of low-arousal negative words during lexical decision in both groups. Interestingly, Zen meditation elicited a global facilitation of all response times, which we discuss in terms of increased attentional resources after meditation.

## Introduction

The scientific interest on meditation has grown substantially over the last two decades, as has our understanding of its neurocognitive mechanisms in emotion regulation (for reviews see [1,2]). While it is well known that behavioral and neural responses to emotional images can be attenuated by meditation [3–5], it is less clear whether meditation also affects higher-level emotional representations such as words. Therefore, the aim of the present study was to examine whether the meditation practice of adept meditators has an impact on emotional valence ratings of word stimuli, as well as on emotional valence effects in visual word recognition [6,7].

### Meditation in attention and emotion regulation

Meditation is the mental practice of increasing attention and calmly observing all experiences in the present moment. Zen meditation is similar to mindfulness meditation [8] and both styles fall under the category of open monitoring meditation. In open monitoring meditation, the practitioner maintains consistent attention and does not focus on a precise thought or object [9]. In the Japanese Soto Zen tradition, which first developed in the early thirteenth-century, meditation usually consists of seated meditation phases (Zazen) and short phases of walking meditation (Kinhin). Practitioners are seated upon a cushion (Zafu) and are facing the wall. During Zazen, meditators keep their eyes half open and the gaze does not focus on any object [10,11].

The achievement of a readiness of mind, which is a smooth, imperturbable and effortless way of thinking, is described by spiritual Zen literature as an aim of meditation. Practitioners are supposed to be “ready for observing things, and to be ready for thinking”. Concentration should always be present in the meditators mind [12]. In fact, meditation practice can facilitate practitioners’ ability to maintain focused attention [13–16]. For example, expert meditators are able to slow down binocular rivalry switching after meditation [17] and demonstrate a decrease of thought distraction and an increase of the present focus [18].

Thoughts, which naturally cross the mind during Zen meditation, are not judged as “good” or “bad” by the meditator. The existence of these thoughts is noticed and then the practitioner lets them pass by [11]. This practice seems to have a positive effect on the ability to regulate negative emotions. Practitioners can regulate negative emotions such as stress [19], anxiety [16,20] or depression more easily [16,21].

Several studies have demonstrated that meditators show a reduced reactivity to emotional images: Desbordes and colleagues [3] investigated the influence of a mindfulness meditation training that was exercised for two hours a week over a period of eight weeks. They found a decrease in amygdala activation during the presentation of emotional images, thus suggesting a decrease of their emotional impact [22]. Moreover, meditators and comparison subjects showed differences in the late positive potential (LPP) between affective and neutral images [5]. LPP amplitudes over frontal scalp regions in response to negative pictures were greater in the comparison group compared to meditation practitioners. Usually, affective images, in comparison to neutral images, provoke greater LPP amplitudes. A third example showing that experienced meditators were less affected by negative images was provided by Ortner and colleagues [4]. They found that participants with more mindfulness meditation experience presented smaller interference from emotional pictures and reported higher psychological well-being. These results suggest that mindfulness meditation can attenuate the emotional reactivity to affective images.

Longitudinal studies also show that meditation interventions can regulate emotions. In a study by Hölzel and colleagues [23] mindfulness meditation practice led to an increase in brain gray matter density in the left hippocampus—a structure associated with emotion regulation [24]. Finally, Chau and colleagues [25] showed that a sample of older people rated negative and positive images more neutral after a compassion meditation intervention. Though the impact of meditation on emotional picture evaluation has been well explored, to our knowledge there is no study investigating the influence of meditation on affective word processing.

### Emotional word recognition

To determine the emotional impact of word stimuli, a standard practice is to collect valence ratings [26–29]. Participants are exposed to a word stimulus and explicitly judge the emotional valence on a 7-point scale ranging from -3 (very negative) through 0 (neutral) to +3 (very positive). These ratings are taken to select stimulus materials for visual word recognition studies. In the lexical decision task (LDT), emotional evaluation is not explicitly required, but occurs implicitly: Letter strings are displayed to participants, who have to decide under time pressure if the strings are words or not [30,31]. While the influence of stimulus properties (like word frequency, sublexical frequency measures, length, valence and many more) on single word recognition is well examined [32–35], little research has focused on the question whether interindividual differences have an impact on single word recognition. For instance, Siegle and colleagues [36] examined the influence of depression on word recognition. Dysphoric and non-dysphoric subjects conducted a valence identification task, in which words were to be classified as negative, neutral or positive, as well as an LDT. In comparison to non-dysphoric participants, dysphoric subjects identified the valence of positive words significantly slower than the valence of negative words. In the LDT, dysphoric participants recognized negative words slower than neutral words. Non-dysphoric subjects responded significantly faster to negative compared to neutral words. Baldwin and colleagues [37] examined whether subjects’ self-confidence could influence their RTs to emotional words. Secure participants were faster than insecure subjects to recognize words which expressed positive interpersonal outcomes. In contrast, insecure subjects identified words expressing negative interpersonal outcomes faster. In sum, there are several studies showing that interindividual differences have an impact on emotion effects in the lexical decision task [38]. There is no study, however, that addresses the emotion-regulation capabilities of meditators in word stimuli.

### The present study

The present work examined whether Zen meditation has an influence on the processing of single words. Adept Zen practitioners and age- and gender-matched comparison subjects conducted an LDT with positive, neutral, and low- and high-arousal negative word stimuli before and after a meditation or comparison intervention. After the LDTs, they rated the valence of the respective words. We used the stimulus set of Hofmann and colleagues [6]. For the comparison group, we expected no difference between the valence ratings before and after the comparison intervention. We also assumed that, if the meditation session has an impact on emotion regulation in the Zen group, the meditation treatment might act to decrease the emotional valence ratings.

For the LDT, we expected to replicate the results of Hofmann and colleagues [6] in the comparison group. They found that low-arousal negative words were processed slower than neutral words. Responses to positive and high-arousal negative words were faster than the responses to neutral words. We also follow these authors in terms of sample size (N = 20; see also [26], for valence ratings with the same sample size). For the meditation group, the critical question was whether the meditation treatment had an influence on the emotional experience as reflected in outcome measures of the LDT. An impact on emotion regulation by meditation would be evident in diminished effects of word affectivity on outcome measures of the LDT after meditation as compared to before meditation. If the meditation session, in contrast, has an unspecific effect on attention capacities, generally faster response times may be observed after, as compared to before meditation. To control interindividual differences between both groups irrespective of meditation, we further assessed concentration capacity, intelligence and personality traits of all participants to use them as potential covariates.

## Method

### Participants

The Zen meditation group consisted of twenty German native speakers (9 female, 19–53 years of age, mean age = 33.3, SD = 11.13). They were recruited at local meditation centers in North Rhine-Westphalia, Germany and had a meditation experience of M = 7.9 years (0.5–28 years, SD = 4.9 years). The weekly meditation practice amounted to M = 4.3 hours (SD = 1.6 hours). The average age and gender of twenty German native speakers was matched for the comparison group (9 female, 18–56 years of age, mean age = 34.5, SD = 11.26). Participants of the comparison group were recruited at the University of Wuppertal or via online advertisements. All subjects of the comparison group reported no prior meditation or yoga experience. The subjects received course credit or money and sweets for their participation. All subjects of the present study gave informed written consent.

This work was carried out in accordance with the ethical standards provided by the Declaration of Helsinki and the German Society for Psychology (DGPs). The Ethics Committee of the University of Wuppertal granted approval for our study.

To take into account interindividual differences in concentration capacity, intelligence and personality traits all subjects conducted three covariate tests. First, participants completed the d2-Revision test, which measures concentration and attention ability. This time-pressured assessment requires participants to cross out the letter “d” with two marks and ignore similar looking distractors in a continuing row of characters [39]. Second, participants were asked to discriminate increasingly complex target words from nonword distractors in the Multiple-Choice Vocabulary Intelligence Test (MWT-B) [40]. Third, participants completed the Big Five personality test (B5T). Originally the test had five scales “neuroticism” (Cronbach’s alpha, α = .90), “extraversion” (α = .87), “conscientiousness” (α = .77), “agreeableness” (α = .76) and “openness to experience” (α = .76). We used the revised version, which has been extended by the three basic requirements “need for safety and peace” (α = .84), “need for power and influence” (α = .78) and “need for achievement and performance” (α = .82) [41].

### Materials

Our stimulus set was composed of 200 German nouns and 200 nonwords. We used the stimulus set from Hofmann and colleagues [6], including the stimulus conditions of high-arousal negative, low-arousal negative, neutral and (low-arousal) positive words. The Berlin Affective Word List Reloaded (BAWL-R) provided too few high-arousal positive nouns (see Fig 1 in [26]) to generate this condition while likewise matching for nine other variables known to affect word recognition (cf. [6]). To prevent undesired influences of word properties, words were carefully matched for emotional valence, arousal, imageability, number of letters, number of syllables, word frequency, number of orthographic neighbors, mean letter frequency (type), mean letter frequency (token), mean bigram frequency (type) and mean bigram frequency (token) (see Table 1 in [6], for stimulus characteristics). The 200 nonwords were built by replacing the vowel of a word with either another vowel or with a consonant. To generate two comparable stimulus sets for the pre- and post-tests, the stimuli of the experimental conditions were divided into subset A and subset B. We conducted 4 x 2 ANOVAs for the control variables to make sure that the subsets did not differ with respect to the control variables (all F’s < 1). In total, every subset consisted of 100 words and 100 nonwords. Subset A and B were used as pre- and post-test in half of the participants and for the other half vice versa.

### Procedure

#### Pre-test

**Fig 1:**
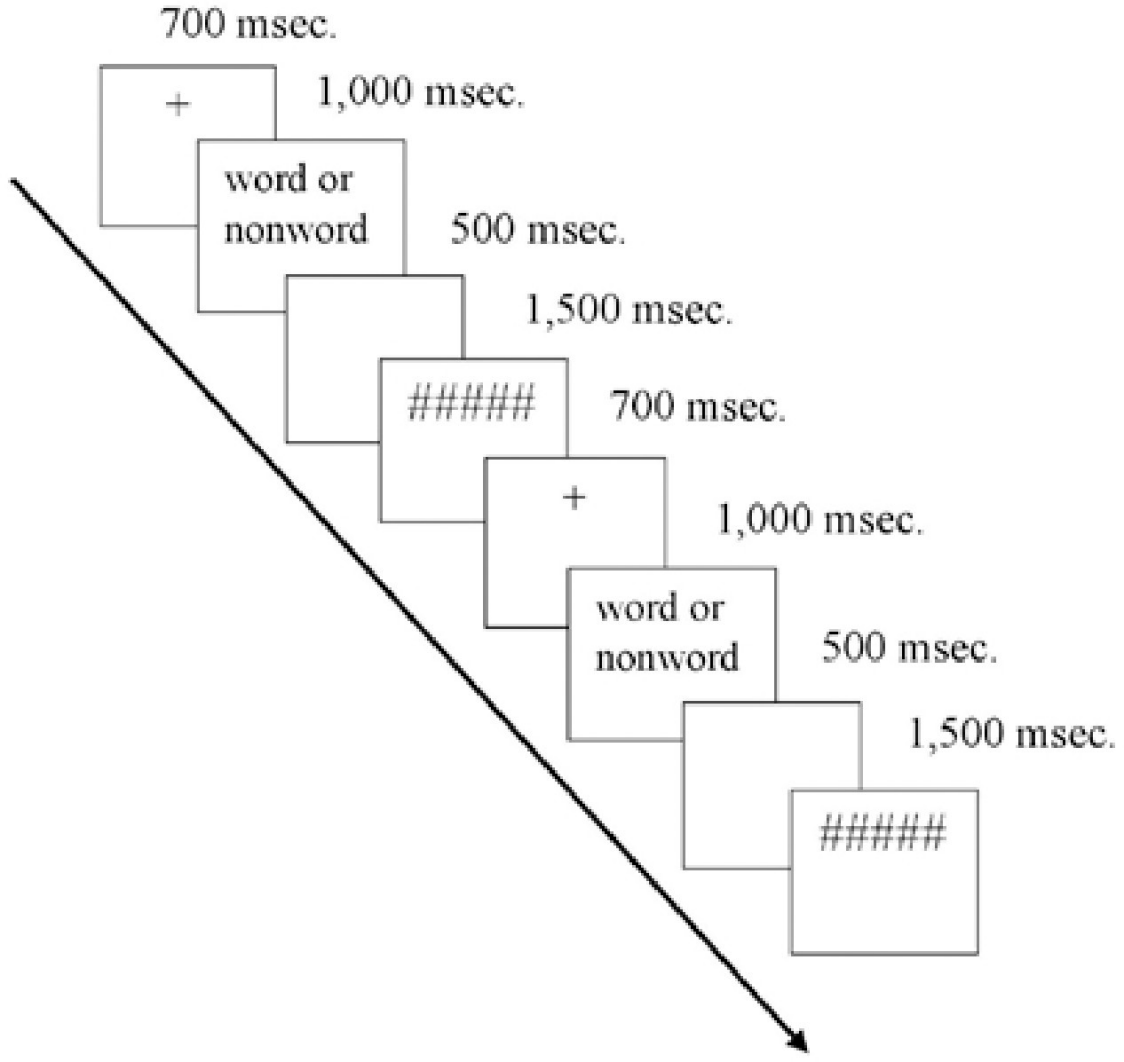
Experimental timeline for the LDT experiments.

After addressing the interindividual differences (see Participants for more details on psychometric assessments), participants started with the main experiment. They sat in front of a 17-in. color monitor (70 Hz). The laboratory was dimly illuminated. Eye-monitor distance was about 65 cm. Subjects were instructed to press a button with the left index finger for nonwords and another button with the right index finger for words. All participants were instructed to respond as fast as possible without reducing accuracy. Five practice stimuli preceded each LDT. Stimuli were displayed on a white screen in black uppercase letters (Times New Roman font, 20 pt). The software PsychoPy was used for stimulus presentation (version 1.82.01, [42]). Stimulus order was pseudorandomized, such that no more than three words or nonwords appeared consecutively. In each trial, a fixation cross (+) was displayed for 700 ms, then the stimulus was shown for 1,000 ms. To put the participants under severe time pressure, they were required to respond within this time window (c.f.[6]). Then, a blank screen was presented for 500 ms, followed by a mask (#####) for 1,500 ms (see Fig 1). After a break of approximately five minutes, participants performed a valence rating on the words presented in the LDT. The seven-point rating scale ranged from -3 to 3 (-3 = very negative, 0 = neutral, 3 = very positive).

#### Meditation and comparison interventions

The 1.5-hour meditation session consisted of two 25-minute seated Zen-Meditations (Zazen) followed by 5-minute walking Zen-Meditations (Kinhin). Additionally, spoken mantras were recited for approximately 30 minutes, for instance the heart sutra representing the Buddhist principle of emptiness [43]. The participants of the comparison group watched a 90-minute interesting but calm and non-arousing documentary landscape movie called “Unsere Erde” (“Our Earth”, [44]).

*Post-test*. After the meditation or comparison intervention, subjects participated in the post-test of the experiment. The LDT procedure was the same as for the pre-test.

## Data analyses

From the valence rating task, we obtained answers on a seven-point rating scale from -3 to 3. We recorded the RTs of the correct answers from the LDT. For the statistical analyses of the RT data and for the valence-rating data, we calculated linear mixed effects models (LMEs) using the lmer function from the lme4 library (version 1.1–14, [45]) in the statistical software environment R [version 3.4.2, http://cran.r-project.org]. An LME analysis allows considering subject and item variance in a non-hierarchical approach [46]. We fitted „subjects” and „items” as random effects, and “group” (meditators/comparison), “time” (before/after) and “emotional valence” (high-arousal negative/low-arousal negative/positive words) as fixed effects. For the representation of the fixed effects, we used effects-coding; high-arousal negative, low-arousal negative, and positive words were contrasted against neutral words. Separate LME analyses were calculated for the Zen and for the comparison group to examine significant interactions. Normality of the residuals was examined using qqplots and the empirical distributions. For the valence-rating model, data points outside the range of -3 and 3 SD of the residual error of the model were rejected from the analyses, which eliminated less than 1% of the data. RTs were log-transformed. We report p-values based on the Satterthwaite approximation using the lmerTest package (version 2.0–36, [47]).

## Results

### Interindividual differences

We tested participants of both groups for significant differences in intelligence, concentration capacity and personality traits to control the influence of these traits on RT and valence rating results (cf. Table 1). Because “openness to experience” was higher and “need for achievement and performance” was lower in the Zen meditation group, we inserted them as covariates in the valence rating and LDT analysis.

**Table 1:** Statistical group differences, mean values and standard deviations of covariate tests.

Covariate Test	Statistical Group Differences	Zen Group		Comparison group	
Big Five personality test		*M*	*SE*	*M*	*SE*
Neuroticism	t(38) = -0.71, p = 0.48	4.10	0.32	4.40	0.28
Extraversion	t(38) = 0.20, p = 0.84	5.80	0.34	5.70	0.36
Conscientiousness	t(38) = 0.39, p = 0.70	4.75	0.35	4.55	0.37
Agreeableness	t(38) = 0.15, p = 0.88	5.25	0.39	5.15	0.53
Need for Safety and Peace	t(38) = 1.74, p = 0.09	4.30	0.31	5.15	0.38
Need for Power and Influence	t(38) = -1.90, p = 0.06	4.55	0.36	5.35	0.22
Openness	t(38) = 3.36, p = 0.01	6.35	0.36	4.45	0.44
Need for Achievement and Performance	t(38) = -2.35, p = 0.03	4.00	0.33	5.20	0.39
Concentration Capacity Test (d2-R)		*M*	*SE*	*M*	*SE*
Number of Processed Target Objects	t(38) = -0.11, p = 0.92	168.25	6.43	169.20	6.39
Concentration Capacity	t(38) = 1.04, p = 0.31	150.78	4.61	141.20	7.99
Percentage of Errors	t(38) = -1.36, p = 0.18	11.86	1.71	15.59	2.14
Intelligence Test (MWT-B)		*M*	*SE*	*M*	*SE*
	t(38) = -0.13, p = 0.90	24.95	0.92	25.10	0.73

### Valence rating and lexical decision data

The overall analysis of the valence rating and the lexical decision data revealed significant main effects of “time” and significant interactions of “time” and “group” (see Table 2 for the overall analysis). In the valence rating analysis, “high-arousal negative” and “low-arousal negative”, as well as “positive” words provided significant two-way interactions with “time”, and three-way interactions with “time” and “group”. Moreover, we found a significant main effect of “high-arousal negative” words in the rating data. In the lexical decision analysis, we found a significant main effect of “low-arousal negative” words.

**Table 2:** Estimates of the regression coefficients, their standard errors, t-values and p-values for the valence and the LDT experiments in the overall analyses.

	Valence Ratings				Lexical Decision Task			
	B	SE	t	p	B	SE	t	p
Time	0.16	0.026	5.99	0.001 ***	-0.06	0.004	-13.70	0.001 ***
Group	-1.12	0.181	-0.95	0.319	0.03	0.549	0.06	0.956
High-arousal negative	-1.66	0.845	-1.97	0.049*	-0.01	0.008	-0.41	0.685
Low-arousal negative	-1.31	0.841	-1.56	0.118	0.02	0.009	1.97	0.049*
Positive	1.07	0.843	1.27	0.206	-0.01	0.009	-1.34	0.182
Time:Group	-0.15	0.037	-3.89	0.001***	0.05	0.006	8.92	0.001***
Time:High-arousal negative	0.25	0.046	5.58	0.001***	-0.01	0.008	-1.11	0.266
Group:High-arousal negative	1.16	1.178	0.98	0.325	-0.01	0.008	-0.45	0.649
Time:Low-arousal negative	0.23	0.046	5.04	0.001***	0.01	0.007	0.89	0.374
Group:Low-arousal negative	0.99	1.176	0.84	0.401	0.01	0.007	1.38	0.168
Time:Positive	-0.36	0.046	-7.87	0.001***	-0.01	0.007	-0.02	0.986
Group:Positive	1.24	1.176	1.06	0.291	0.01	0.007	0.34	0.732
Time:Group:High-arousal negative	-0.28	0.065	-4.33	0.001***	-0.01	0.011	-0.26	0.822
Time:Group:Low-arousal negative	-0.19	0.065	-3.01	0.003**	-0.01	0.011	-0.66	0.508
Time:Group:Positive	0.36	0.065	5.52	0.001***	-0.01	0.011	-0.37	0.713
Openness	0.01	0.023	0.46	0.647	0.06	0.138	0.40	0.691
Need for Achievement and Performance	-0.03	0.026	-1.18	0.244	0.01	0.025	0.57	0.568

“***” = p < 0.001

“**” = p < 0.01

“*” = p < 0.05

To resolve the interactions with the factor “group”, we fitted separate LMEs for the Zen and the comparison group (cf. Table 3). Here, we found significant valence rating and lexical decision effects of “time” for the Zen group, but not for the comparison group. This indicates that the meditation session had an effect on meditators, but the comparison intervention did not affect our comparison group.

**Table 3:** Estimates of the regression coefficients, their standard errors, t-values and p-values for the LDT in the Zen and comparison group.

	Valence Ratings				Lexical Decision Task			
	B	SE	t	p	B	SE	t	p
**Zen Group**								
Time	0.15	0.026	5.91	0.001***	-0.06	0.004	-14.04	0.001***
High-arousal negative	-1.84	0.838	-2.19	0.028*	-0.01	0.008	-0.42	0.675
Low-arousal negative	-1.51	0.835	-1.81	0.071	0.02	0.008	1.99	0.046*
Positive	0.88	0.837	1.05	0.295	-0.01	0.007	-1.47	0.141
Time:High-arousal negative	0.25	0.045	5.48	0.001***	-0.01	0.007	-1.14	0.252
Time:Low-arousal negative	0.23	0.045	5.14	0.001***	0.01	0.007	1.07	0.284
Time:Positive	-0.36	0.045	-7.85	0.001***	-0.01	0.007	-0.11	0.917
Openness	0.07	0.040	1.71	0.104	0.07	0.228	0.29	0.768
Need for Achievement and Performance	-0.04	0.043	-0.94	0.361	0.01	0.245	0.03	0.975
**Comparison Group**								
Time	0.01	0.027	0.39	0.691	-0.01	0.004	-1.09	0.275
High-arousal negative	-0.28	0.861	-0.32	0.748	-0.01	0.009	-0.69	0.489
Low-arousal negative	-0.09	0.856	-0.12	0.915	0.03	0.009	2.94	0.004**
Positive	2.55	0.859	2.97	0.003**	-0.01	0.009	-0.99	0.321
Time:High-arousal negative	-0.02	0.046	-0.34	0.735	-0.01	0.007	-1.51	0.131
Time:Low-arousal negative	0.04	0.046	0.83	0.407	0.01	0.007	0.09	0.922
Time:Positive	-0.01	0.046	-0.27	0.791	-0.01	0.007	-0.47	0.639
Openness	-0.03	0.024	-1.13	0.271	0.05	0.178	0.26	0.801
Need for Achievement and Performance	-0.03	0.027	-1.01	0.270	0.01	0.026	0.48	0.632

“***” = p < 0.001

“**” = p < 0.01

“*” = p < 0.05

**Fig 2:**
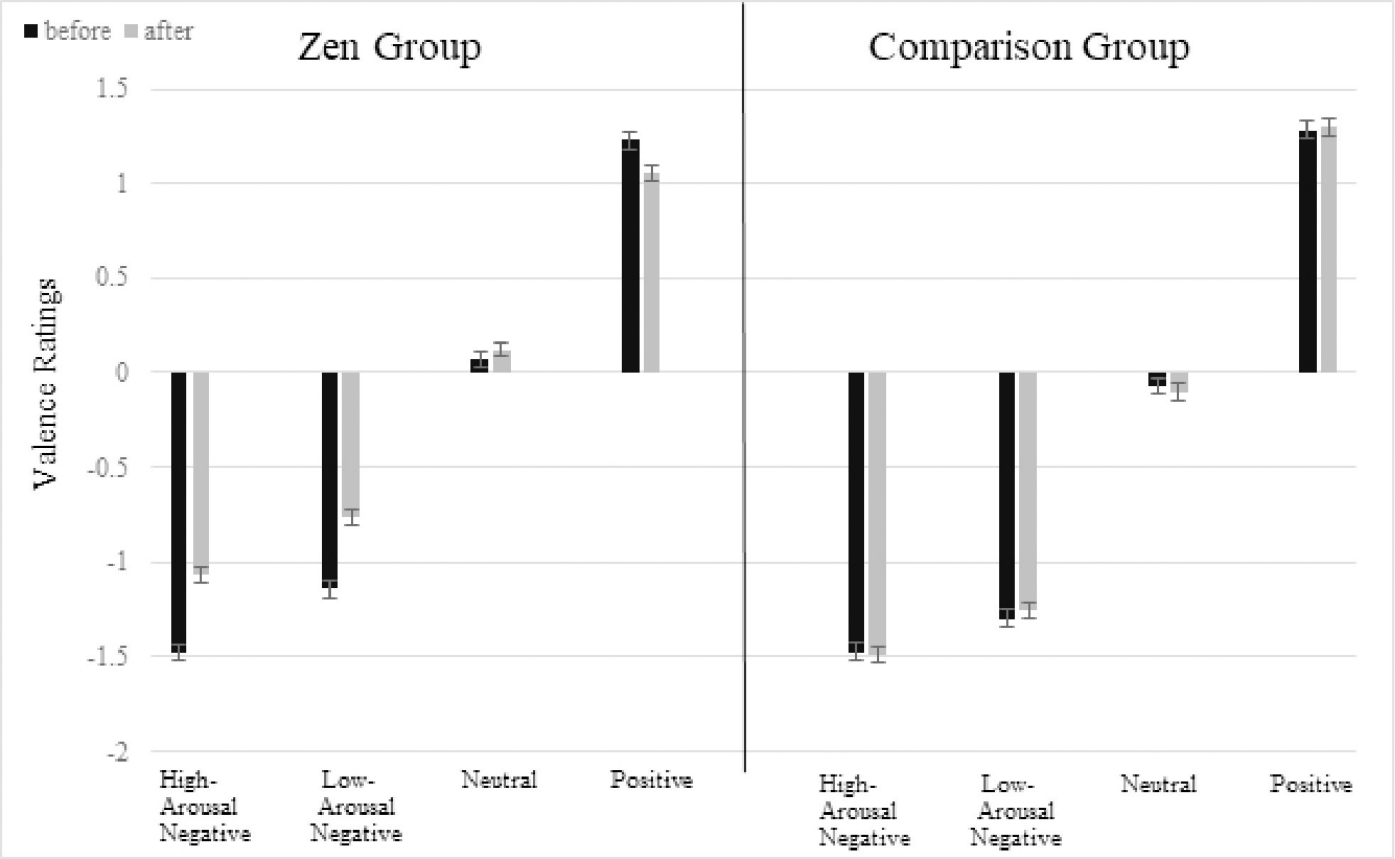
Valence rating results. Error bars indicate standard errors.

In the valence rating analysis of the Zen group, there were significant two-way interactions of “time” with “positive”, “high-arousal negative” and “low-arousal negative” words. Fig 2 shows that emotional valence ratings became more neutral after the Zen meditation session. When examining the main effect of high-arousal negative words from the overall analysis in detail, we found no significant effect in the comparison group, but a significant effect in the Zen group. We speculate that this in part results from the “neutral” words, which were rated slightly positive in the Zen group and slightly negative in the comparison group, resulting in a smaller difference (cf. Fig 2). This negativity in ratings for neutral words may also account for the effect of positive valence in the comparison group.

**Fig 3:**
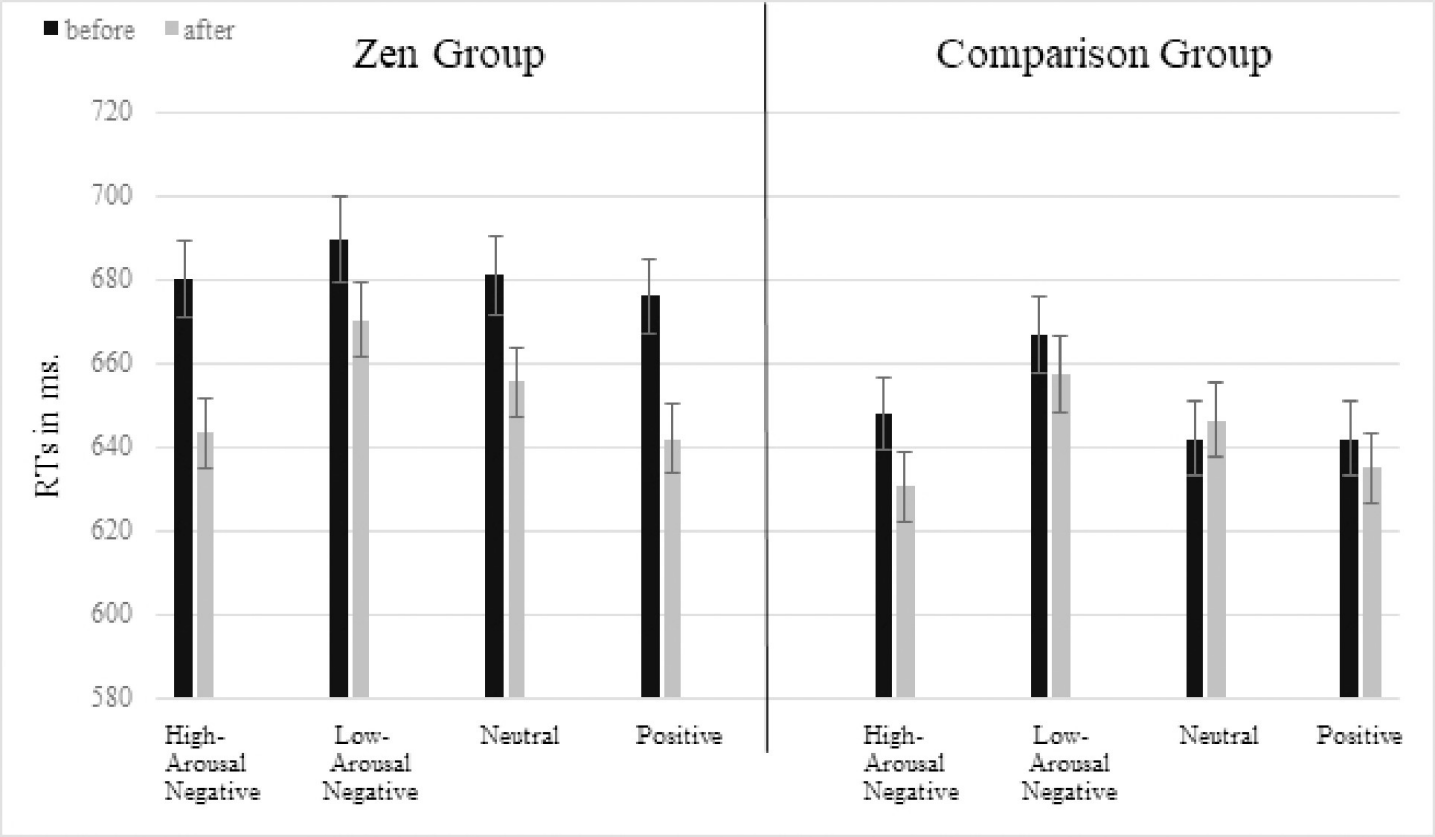
RTs in the lexical decision experiment. Error bars indicate standard errors.

Fig 3 shows that the lexical decision effect of “time” in the Zen group resulted from an unspecific facilitation due to the meditation session. The descriptive data might appear to suggest that meditators demonstrated, before the interventions, generally slower RTs than comparison subjects did. However, response times do neither differ significantly between groups at baseline (b = -0.06, SE = 0.481, t = -0.12, p > 0.05), nor after the interventions (b = -0.01, SE = 0.486, t = -0.01, p > 0.05). Finally, the lexical decision main effect of low-arousal negative words was replicated in the Zen and in the comparison group, respectively (cf. [6]). To assure the robustness of our results, we computed the statistical models without any covariates. All significant effects remained the same. When we calculated the analysis with the marginally significant covariates “need for power/influence” and “need for safety/peace” significant effects did not change either.

## Discussion

Our sample of Zen meditators showed larger “openness to experience” and less “need for achievement and performance” than the comparison group. In order to control these traits’ effects on word processing outcomes, we included them as covariates in the analyses. After a 90-minute Zen meditation, valence ratings were more neutral than before, while the comparison intervention elicited no change of valence ratings. Even though the explicit evaluation of words was affected by meditation, we found no effects of meditation in the lexical decision task, where affective evaluation was not explicitly required. Rather, we were able to replicate the effect of low-arousal negative words during lexical decision in both groups [6]. Finally, word recognition was accelerated by Zen meditation compared to performance before the meditation intervention. The comparison group showed no significant differences in lexical decision RTs after comparison intervention.

### Valence rating experiment

While previous studies revealed that meditation can reduce the emotional impact of picture stimuli [e.g. 3,22], the present study shows that meditation also has a substantial effect on emotional word processing. Adept meditators rated the valence of high- and low-arousal negative and low-arousal positive words as more neutral after the meditation intervention. Emotion regulation, elicited by the meditation intervention, could be an explanation for the neutralized valence ratings. During Zen meditation, practitioners train not to judge thoughts on a positive-negative scale [11]. In fact, several studies show that meditation practice can help to regulate practitioners’ emotions. Tang and colleagues [16] demonstrated lower depression and anger scores as well as a significant decrease in stress-related cortisol after 5 days of meditation training. Meditation practice can lead to a decreased sensation of anxiety [see 20, for a review] and stress [see 19, for a review]. Moreover, after an 8-week mindfulness meditation training, the amygdala of meditators was less activated during the display of emotional images than before the meditation training [3]. Our findings contribute to the discussion of a role of meditation in emotion regulation by demonstrating a potential mechanism: Meditation may help to down-regulate emotions and dampen the emotional assessment of word stimuli.

While we report neutralized emotional states being associated with more neutral valence ratings, Kanske and Kotz [48] found in a complementary study that increased emotional states, such as anxiety and depression, increased emotional valence ratings of words: In their study, subjects with greater depression scores displayed more negative valence ratings of negative and positive words. Participants with higher anxiety scores rated negative words as more arousing and more negative, thereby corroborating the notion of emotional states influencing stimulus valence ratings.

### Lexical decision task

While we found emotion regulation effects in the explicit valence rating task, there was no evidence suggesting that meditation also changed the implicit effects of affective word features in the LDT. We replicated the results of Hofmann and colleagues [6] in both, the experimental and the comparison groups, indicating that low-arousal negative words are processed generally slower than neutral words. We were not able to replicate their effects that high-arousal negative and (low-arousal) positive words were processed faster than neutral words, even though there was a tendency in this direction after the meditation and comparison intervention in both groups. Electrode preparation in the EEG study by Hofmann and colleagues’ [6] might have elicited a moderate level of physical stress for the participants that “boosted” positive and high-arousal negative word effects such that the sample size of 20 was enough to detect statistical effects. Examining the exact t-values in our study, however, did not hint towards a tendency of these main effects, suggesting that these missing findings were not a matter of sample size.

Hofmann and colleagues [6] were targeting very early ERP effects of affective word features around 100 ms after stimulus presentation. Therefore, participants were put under severe time pressure by forcing them to respond within one second (see [6] for a thorough discussion). Hofmann and colleagues [6] found no effects of emotional valence on the LPP available in emotional face or word stimuli [49]. Therefore, we suggest that the present, very similar experimental paradigm resulted in a de-emphasis of later, more evaluative stages of processing [5]. To test for this hypothesis, future studies should explore Zen effects on affective word processing with lower time pressure using an experimental task emphasizing late evaluative processes [50].

Rather than finding emotion-specific effects of Zen, we showed that a 90-minute meditation session leads to generally faster word recognition in the LDT. The RTs of the comparison subjects did not change after the comparison intervention. A number of neurocognitive mechanisms might be instrumental in explaining these results.

First, meditation practice supports emotion regulation [3,16,19,20]. We assume that due to regulated emotions meditators, in the present study, participants were less distracted by the emotional content of word stimuli and therefore able to provide generally faster responses in the LDT. The reverse effect may be present in depressed participants. Siegle and colleagues [36] found that in a LDT dysphoric subjects recognized negative and positive words significantly slower than neutral words. In this case, increased emotions may have distracted participants, slowing down word processing.

Second, meditation practice may lead to the acceleration of RTs, because of an augmentation in practitioners’ focused attention. Several experimental studies support this view: Slagter and colleagues [15] found that a 3-month intensive meditation practice can reduce the attentional-blink deficit: When two target stimuli are presented in rapid succession, the second target stimulus is often not consciously recognized. In a study by Tang and colleagues [16], a group of novices practiced meditation for five days and a comparison group participated in a relaxation training for the same amount of time. The meditation group displayed significantly better results in the Attention Network Test (ANT). Chan and colleagues [13] showed that meditation practice leads to reduced interference in the Stroop task, which suggests that meditation experience might increase the efficiency of the executive attentional network. Finally, in a study by Pagnoni and Cekic [14], comparison subjects showed a negative correlation of both gray matter volume and attentional performance with age, which was not evident for Zen practitioners. In the context of this literature, our globally facilitated RTs to word stimuli may well be explained by the improved attentional resources after Zen meditation.

Third, meditation practice may have accelerated RTs because of reduced mind-wandering. In a study by Brefczynski-Lewis and colleagues [51], expert meditators demonstrated decreased brain activation in regions of the default mode network, which is associated with discursive thoughts. Pagnoni and Cekic [52] also showed that meditation practice can reduce the neural activity associated with conceptual processing in default mode network regions. The authors suggest that meditation practice can support the control of automatic cascades of semantic associations and therefore facilitate the regulation of spontaneous mind-wandering. These processes might have helped Zen practitioners in the present study to stay focused on the presented word stimuli and thereby facilitated their responses.

## Conclusions

The present work is the first to demonstrate that Zen meditation can lead to neutralized valence ratings of affective words. We assume that emotion regulation, elicited by the meditation intervention, is primarily responsible for this effect. We found no differential emotion effects during lexical decision in the Zen vs. comparison group. While participants in the valence rating task have no real time constraints for an explicit emotional evaluation of the stimuli, affective information can only implicitly act on lexical decision performance. Moreover, time pressure in this experiment may have fostered an early affective-semantic evaluation that is not affected by meditation. Even though meditation did not influence the emotion effects during lexical decision, participants demonstrated globally faster word recognition after meditation. We suggest that greater attentional resources after meditation can best explain this result.
